# Antigenic variation in African trypanosomes: the importance of chromosomal and nuclear context in VSG expression control

**DOI:** 10.1111/cmi.12215

**Published:** 2013-10-10

**Authors:** Lucy Glover, Sebastian Hutchinson, Sam Alsford, Richard McCulloch, Mark C Field, David Horn

**Affiliations:** 1 Division of Biological Chemistry & Drug Discovery, College of Life Sciences, University of DundeeDow Street, Dundee, DD1 5EH, UK; 2 London School of Hygiene & Tropical MedicineKeppel Street, London, WC1E 7HT, UK; 3 Wellcome Centre for Molecular Parasitology and Division of Infection and Immunity, Glasgow Biomedical Research Centre, University of Glasgow120 University Place, Glasgow, G12 8TA, UK

## Abstract

African trypanosomes are lethal human and animal parasites that use antigenic variation for evasion of host adaptive immunity. To facilitate antigenic variation, trypanosomes dedicate approximately one third of their nuclear genome, including many minichromosomes, and possibly all sub-telomeres, to variant surface glycoprotein (VSG) genes and associated sequences. Antigenic variation requires transcription of a single *VSG* by RNA polymerase I (Pol-I), with silencing of other *VSG*s, and periodic switching of the expressed gene, typically via DNA recombination with duplicative translocation of a new *VSG* to the active site. Thus, telomeric location, epigenetic controls and monoallelic transcription by Pol-I at an extranucleolar site are prominent features of *VSG*s and their expression, with telomeres, chromatin structure and nuclear organization all making vitally important contributions to monoallelic *VSG* expression control and switching. We discuss *VSG* transcription, recombination and replication control within this chromosomal and sub-nuclear context.

## Introduction

Control of antigenic variation in pathogens is of interest for at least two major reasons. First, it is clearly an important immune evasion and virulence strategy, and second, it frequently requires monoallelic gene expression, a common mechanism also utilized by mammals, for our sense of smell, for example (Lyons *et al*., 2013). Many pathogenic protists express major and variable surface proteins one at a time for immune evasion, including *Plasmodium falciparum*, the malaria parasite (Guizetti and Scherf, 2013), *Giardia*, the cause of intestinal giardiasis (Prucca and Lujan, 2009), and *Trypanosoma brucei*, the tsetse-fly transmitted African trypanosome that causes sleeping sickness in humans and nagana in livestock. Our knowledge regarding mechanisms that silence all but one gene has improved recently. For example, an H3K36 histone methyltransferase, PfSETvs, is required for *var* gene silencing in *P. falciparum* (Jiang *et al*., 2013), RNA interference is required for *VSP* gene silencing in *G. lamblia* (Prucca *et al*., 2008) and the telomere-binding protein RAP1 is required for *VSG* silencing in *T. brucei* (Yang *et al*., 2009). Our understanding of the mechanisms selecting a single gene for activation is less advanced.

**Figure 1:**
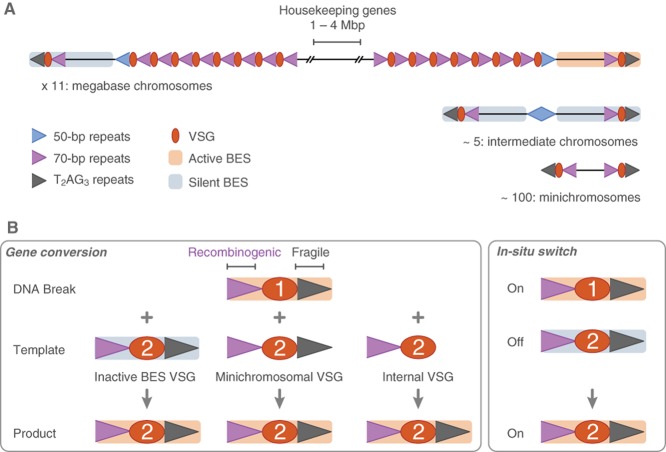
The chromosomal context of *VSG* genes.A. The schematic shows how up to 2000 *VSG* genes and *VSG* pseudo-genes occupy the subtelomeres of three different classes of *T. brucei* chromosomes: megabase, up to 6 Mbp; intermediate, 150–500 kbp; mini, 50–100 kbp. Repetitive sequences found at these loci are thought to be important for monoallelic *VSG* expression control and for *VSG* switching via DNA double-strand break (DSB) repair. Telomeric T_2_AG_3_-repeats cap all *T. brucei* chromosomes. Megabase chromosomes are diploid with hemizygous sub-telomeres. Bloodstream expression site (BES) promoters, *ESAGs* and metacyclic expression sites are not shown.B. Mechanisms of antigenic variation; switching from *VSG1* to *VSG2*. Telomere-proximity is thought to render the active *VSG* locus prone to DSBs. The 70 bp repeats then facilitate gene conversion, replacement of the active *VSG* with a *VSG* (or *VSG* segment) copied from any one of multiple alternative locations. Monoallelic *VSG* expression is also maintained during an *in-situ* transcription switch. Only the relevant portions of the BESs are shown.

**Figure 2:**
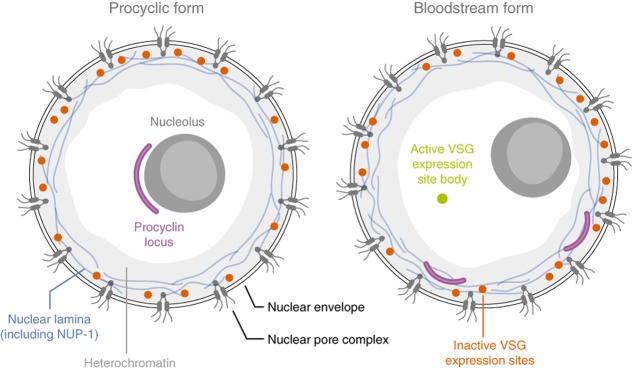
Nuclear sub-compartments in trypanosomes. A procyclic and bloodstream form nucleus is shown, with the region of heterochromatin (corresponding to ∼ 15% of nuclear volume) in light gray. The nucleolus is shown as a dark gray structure within the nucleus, and in the procyclic form is associated with the procyclin loci (purple). Nuclear pore complexes are shown as double lozenges, and the nuclear lamina as a thin blue lines within the region of heterochromatin. Inactive *VSG* expression sites are red and located at the nuclear periphery, while the active locus is in green, and is equivalent to the expression site body (ESB). The major changes are that the procyclin loci are downregulated in the bloodstream form and likely come to reside within the heterochromatin, while a single *VSG* expression site associates with the ESB. While morphologically there may be differences in heterochromatin, evidence is consistent with peripheral chromatin bearing inactive genes in both life cycle stages.

Location is important, and for *VSGs*, both the chromosomal location (Fig. 1) and position within the nucleus (Fig. 2) appear to be critical. In addition, transcription states and chromatin states frequently go hand-in-hand with nuclear position; typically, silent, condensed chromatin occupies peripheral ‘heterochromatic’ space. Sub-telomeres in many organisms are populated with contingency genes, which are often only expressed when needed, and these regions tend to conform to this heterochromatic paradigm. However, in monoallelic expression systems a single active gene escapes the silent heterochromatin. In the case of *T. brucei VSGs*, the escapee is transcribed at a telomere by RNA polymerase I (Pol-I) (Gunzl *et al*., 2003). This single *VSG*, rather than associating with Pol-I at the nucleolus as previously suspected, is held at a distinct extranucleolar site (Chaves *et al*., 1998; Navarro and Gull, 2001). DNA recombination and nuclear dynamics are also important here since *VSGs* can be translocated to the active site to bring about a VSG switch, clearly requiring intimate interactions with the silent archive (Fig. 1B). Transcription can also switch from one telomeric *VSG* to another telomeric *VSG*. Here we focus on *VSG* gene control in African trypanosomes, considering both *cis*-acting sequences and *trans*-acting factors, how they behave in the context of telomeric chromatin and nuclear positioning and how they control *VSG* expression, recombination and replication.

## The telomeric *VSG* environment

The *T. brucei* silent *VSG* archive is located at sub-telomeric loci, with most genes in silent arrays and many *VSGs* immediately adjacent to the telomeres of the megabase, intermediate and minichromosomes (Fig. 1A). The single expressed *VSG* is invariably located immediately adjacent to a telomere in a bloodstream-form expression site (BES) on one of the larger chromosomes (Marcello and Barry, 2007). *VSG* switching is thought to occur with a frequency of 10^−2^/mitotic division. This can decrease ∼ 1000-fold during growth in culture or passage in rodents but returns to the higher rate after passage through the tsetse-fly vector (Turner, 1997), possibly due to erasure of a DNA or chromatin modification (see below). In common with the core *T. brucei* transcriptome, the active BES is transcribed as a polycistronic unit and individual mRNAs are generated by co-transcriptional *trans*-splicing. Unlike most of the genome, however, the BES is transcribed by Pol-I (Gunzl *et al*., 2003). Metacyclic *VSGs*, which normally drive expression of the VSG coat in cells in the tsetse-fly salivary glands, are also transcribed by Pol-I at telomeres, but these *VSGs* are found in monocistronic units (Pedram and Donelson, 1999; Kolev *et al*., 2012). Analysis of 15 *T. brucei* BESs revealed striking similarities, both in sequence and structure (Hertz-Fowler *et al*., 2008). Functional BESs have a telomere-distal RNA Pol-I promoter, an array of ES-associated genes (*ESAGs*) with largely conserved synteny and two promoter-proximal genes (*ESAG6* and *ESAG7*) that encode a novel transferrin receptor (Steverding *et al*., 1994). Many BESs also contain *ESAG* and *VSG* pseudogenes. Indeed, several BESs lack specific functional *ESAGs* and have not been activated *in vitro*, suggesting that a minimum set of *ESAGs* are required for growth. BES *VSGs* are separated from the *ESAGs* by a block of 70 bp repeats and are always adjacent (200–1600 nt spacing) to the telomere repeats (Fig. 1A).

The *T. brucei* telomere comprises T_2_AG_3_-repeats (as also found in humans) of up to ∼ 15 kbp (Horn *et al*., 2000; Dreesen *et al*., 2007) with terminal t-loop structures of ∼ 1 kbp (Munoz-Jordan *et al*., 2001). Telomeres are maintained and extended indefinitely by telomerase reverse transcriptase (TERT) (Dreesen *et al*., 2005; Glover *et al*., 2007), a nucleo-protein complex with an RNA template sequence (Sandhu *et al*., 2013). The transcribed telomere associated with the active BES is extended most rapidly (Horn *et al*., 2000) but may be prone to frequent breaks (Bernards *et al*., 1983; Pays *et al*., 2013; Horn and Cross, 1997). In *TERT*-null mutants, telomere length decreases and, when the length becomes very short (7–27 repeats), TERT-independent stabilization occurs (Dreesen and Cross, 2006a). In these circumstances, the *TERT*-null mutants display elevated *VSG* switching driven by gene conversion (Dreesen and Cross, 2006b; Hovel-Miner *et al*., 2012). It has also been observed that recently isolated strains, thought to switch at a high frequency, have shorter telomeres than observed in the most widely studied ‘slow-switching’ populations (Dreesen and Cross, 2008). A direct test of whether telomere length impacts *VSG* switching in wild-type cells is challenging and has yet to be reported, however.

The *T. brucei* telomere-associated protein complex resembles that described in mammals and yeast, and characterization of the associated factors has yielded important insights into *VSG* expression control. Unexpectedly, many telomere-associated proteins have little impact on *VSG* silencing, including the telomere length regulator Ku70/Ku80 (Conway *et al*., 2002a), the histone H3 variant (Lowell and Cross, 2004), the telomere repeat-binding protein TRF2 (Li *et al*., 2005) or the silent-information regulator SIR2rp1 (Alsford *et al*., 2007). In contrast, RAP1, an essential component of the telomere complex, does play a role in repression of BES *VSGs* (Yang *et al*., 2009). However, further analysis of RAP1-mediated repression showed a more pronounced effect in insect-stage cells and a measurable impact on chromatin structure only in this life cycle stage (Pandya *et al*., 2013). In addition, although telomeres do silence Pol-I transcribed genes in bloodstream-stage *T. brucei*, this effect was restricted to only a few kilobases (Glover and Horn, 2006), and telomere loss at a silent BES fails to derepress that BES (Glover *et al*., 2007). Thus, although current findings support the idea that the telomeric environment is important for *VSG* expression control, with a conserved telomeric silencing mechanism operating in *T. brucei*, as also observed in budding yeast (Pryde and Louis, 1999), additional mechanisms likely contribute to *VSG* silencing in the context of a monoallelic expression system.

## The nucleolus, the ESB and the nuclear periphery

In common with most eukaryotic cells, the trypanosome nucleus is subdivided internally. These intranuclear compartments were originally described by ultrastructure and differential electron density, and while the nucleus is now known to possess multiple domains or compartments, the nucleolus, nuclear envelope and differentiation between high and low electron dense hetero-and euchromatin clearly also applies to trypanosomes (Vickerman and Preston, 1970; Rout and Field, 2001). The nucleolus in trypanosomes, as in other eukaryotes, is the site of *rRNA* transcription and ribosome assembly. Indeed, the nucleolus is rapidly disassembled and morphologically lost following inhibition of transcription with actinomycin D (Nazer and Sanchez, 2011). In trypanosomes, the nucleolus also serves as a location for transcription of the *procyclin* locus (Landeira and Navarro, 2007), encoding the superabundant procyclin antigens at the surface of insect, midgut-stage cells (Roditi *et al*., 1987) (Fig. 2). This location can be rationalized by the need for Pol-I mediated *procyclin* transcription, and the polymerase involved is apparently identical to the Pol-I involved in *rRNA* transcription (Gunzl *et al*., 2003). By direct visualization, *procyclin* genes localize at the nucleolar periphery in procyclic stages (Landeira and Navarro, 2007). Significantly, this peripheral location is also the site of most transcriptional activity as detected by BrUTP-labelling. While formal determination of the location of the *procyclin* locus in bloodstream cells is lacking, its absence from the nucleolar periphery and incorporation into peripheral heterochromatin can be inferred by derepression following knockdown of the lamina protein NUP-1 (DuBois *et al*., 2012).

An apparently unique feature of African trypanosomes is the presence of a developmentally regulated extranucleolar Pol-I containing focus; on account of association with the active *VSG* BES, this structure is called the ES body (ESB) (Navarro and Gull, 2001). The ESB contains only the active BES, while inactive BESs, and hence *VSGs*, presumably occupy a more peripheral heterochromatin compartment (Fig. 2). The ESB is present at all cell-cycle stages in bloodstream-form cells but has never been observed in the insect stage. Indeed, during *in vitro* differentiation, the ESB is lost and the active BES relocates to the nuclear periphery (Landeira and Navarro, 2007). ESB replication/separation is a somewhat late event in mitosis and is important for the stable inheritance of *VSG* expression status; the ESB remains as a single structure into G_2_ and separates later than the *rRNA* genes or the inactive BESs (Landeira *et al*., 2009). When the ESB does segregate into each daughter nucleus, this inheritance requires cohesion, as knockdown of cohesin subunits leads to an increase in switching frequency among BESs (Landeira *et al*., 2009).

The nuclear envelope provides a boundary for the separation of nuclear and cytoplasmic functions, and also a platform for organizing the nuclear interior. Organizing structures include the nuclear pore complex (NPC), the SUN/KASH domain proteins or LINC complex, a large disparate group of spectrin-repeat-containing proteins called Nesprins (Rajgor and Shanahan, 2013), plus the nuclear lamina. The level of conservation of these features between trypanosomes and mammals is remarkably variable. The NPC appears rather well conserved, at least in terms of overall composition (DeGrasse *et al*., 2009), but the LINC complex appears to be absent and there is little evidence for conservation of the other nesprins (Field *et al*., 2012). The lamina is also divergent, so that the 60 kDa intermediate filament proteins of mammals, lamins, are replaced in trypanosomes by a giant, repetitive protein, NUP-1, which is likely a component of fibres at the nuclear envelope (Rout and Field, 2001; DuBois *et al*., 2012). NUP-1 is a major structural protein which seems to assume many of the functions described for lamins in mammals, encompassing structural integrity of the nucleus, NPC positioning and regulation of gene expression. Significantly, knockdown of NUP-1 leads to derepression of inactive BESs, suggesting that the inactive BESs are indeed within heterochromatin, and that NUP-1 acts as an organizer of this region of the nucleus.

## Chromatin and *VSG* expression control

The *T. brucei* histones are divergent with respect to the highly conserved histones of yeasts and metazoans (Alsford and Horn, 2004) and this is particularly true of their *N*-terminal tails. In spite of this divergence, multiple sites of acetylation and methylation on *T. brucei* histones have been identified (Janzen *et al*., 2006a; Mandava *et al*., 2007) and some of these, based on enrichment at probable transcription start-sites, have been implicated in the control of transcription by RNA Pol-II (Siegel *et al*., 2009; Wright *et al*., 2010). Trypanosomes also possess several histone variants, including H3V (Lowell and Cross, 2004), and a DNA base modification, J (β-d-glucosyl-hydroxymethyluracil), both enriched at telomeres (van Leeuwen *et al*., 1997). Many histone modifications have not yet been investigated in any detail but, at this stage, it remains possible that no histone *N*-terminal tail modification, nor any histone variant, nor base-J, play any major role in *VSG* expression control.

Despite this, other aspects of chromatin structure and reversible histone modification do clearly contribute to the control of monoallelic *VSG* expression and antigenic variation in *T. brucei* (Figueiredo *et al*., 2009). Depletion of core nucleosomal histone H3 (Alsford and Horn, 2012) or ‘linker’ histone H1 (Povelones *et al*., 2012) results in derepression of silent BES promoters. A similar effect is also seen upon depletion of the histone chaperones, FACT (Denninger *et al*., 2010), NLP (Narayanan *et al*., 2011), ASF1A or CAF-1b (Alsford and Horn, 2012) and depletion of these chaperones results in different cell cycle defects, reflecting distinct DNA replication-dependent and-independent roles. Depletion of the chromatin remodeller, ISWI, also results in BES promoter derepression, and additionally causes some derepression of silent *VSGs*, though expression remains several orders of magnitude lower than seen at the active *VSG* BES (Stanne *et al*., 2011). Thus, reduction in the number of nucleosomes or changes in their organization at a repressed BES elicits some transcriptional derepression. These findings demonstrate an important role for chromatin in maintaining *VSG* silencing, and also suggest, as proposed earlier (Vanhamme *et al*., 2000), that transcription elongation rather than initiation is repressed.

The location of *VSG* BESs proximal to telomeres suggested that classical sirtuin-dependent telomeric silencing might be involved in monoallelic *VSG* expression. However, though *T. brucei* SIR2rp1, HAT1 and DAC1 influence expression of telomere-proximal reporters, these factors appear to have little or no impact on *VSG* silencing (Alsford *et al*., 2007; Kawahara *et al*., 2008; Wang *et al*., 2010). Instead, two other histone modifying enzymes, DOT1B and DAC3, do impact BES transcription. DOT1B is a dispensable methyltransferase responsible for H3K79 tri-methylation (Janzen *et al*., 2006b), loss of which leads to partial derepression of silent *VSG*s and a significant slowing in switching between BESs (Figueiredo *et al*., 2008). Depletion of DAC3, an acetyltransferase with unknown target(s), results in reporter gene expression from a silent BES promoter, again with no detectable expression of the distal *VSG* (Wang *et al*., 2010).

The active BES escapes repression and is depleted of nucleosomes (Figueiredo and Cross, 2010; Stanne and Rudenko, 2010), but it is unclear whether this is cause or consequence of Pol-I transcription. TDP1, a high mobility group protein, is enriched at actively transcribed Pol-I loci in *T. brucei*, and its depletion results in reduced transcription from *rDNA* loci and the active BES (Narayanan and Rudenko, 2013). These findings led to the hypothesis that TDP1 replaces histones in regions transcribed by Pol-I, thereby maintaining an open chromatin conformation that is amenable to transcription. *T. brucei* ELP3b, a putative acetyltransferase component of the so-called elongator complex, localizes specifically to the nucleolus and attenuates transcription at *rRNA* loci (Alsford and Horn, 2011). To date, no factor that localizes specifically to the ESB has been reported, however, so we do not fully understand the differences between Pol-I transcription in the ESB and in the nucleolus.

## DNA double-strand-break repair and the telomeric environment

Central to antigenic variation is the ability of *T. brucei* to switch the active *VSG*, most commonly by gene conversion (Robinson *et al*., 1999), which requires the conserved DNA repair pathway of homologous recombination (HR). BES *VSGs* are flanked on the telomere distal-side by long stretches of 70 bp repeats, while most archival *VSGs* are flanked by short stretches of similar repeats (Fig. 1); it is these sequences that most frequently mediate *VSG* recombination (Marcello and Barry, 2007). Telomeric (including minichromosomal) *VSGs* predominate as silent donor sequences (Robinson *et al*., 1999) due to greater availability of flanking homologous sequences (Morrison *et al*., 2005). Most likely this explains why *ESAGs* and other BES sequences can also be exchanged via recombination elsewhere in the BES (Hertz-Fowler *et al*., 2008; Boothroyd *et al*., 2009). However, later in an infection, when the telomeric *VSG* archive is exhausted, subtelomeric array *VSGs* come to predominate, with segmental *VSG* conversion producing novel mosaic *VSGs* (Hall *et al*., 2013).

Facilitating studies on HR in *T. brucei*, the yeast meganuclease, I-SceI, has been used to introduce single, locus-specific DNA double-strand breaks (DSBs) (Glover *et al*., 2008). At a chromosome-internal locus, these breaks trigger accumulation of RAD51 foci, a G_2_/M DNA-damage checkpoint and repair predominantly by HR (Glover *et al*., 2008). In addition, I-SceI mediated breaks at the active BES (upstream of the *VSG* and adjacent to the 70 bp repeats) trigger *VSG* switching, suggesting that these breaks mimic the natural triggers for switching (Boothroyd *et al*., 2009; Glover *et al*., 2013). In this case, a break-induced replication mechanism was observed, involving recombination initiated at the 70 bp repeats and duplication of the donor locus to the chromosome end (Boothroyd *et al*., 2009). Notably, BESs display a higher frequency of double-strand breaks relative to a chromosome-internal locus (Glover *et al*., 2013) suggesting that *T. brucei* telomeres render adjacent loci ‘fragile’.

As observed at a chromosome-internal locus, I-SceI mediated DSBs adjacent to the 70 bp repeats at active BESs also trigger a ‘classic’ DSB response with DNA resection, accumulation of γH2A and evidence for a G_2_/M checkpoint (Glover *et al*., 2013); γH2A is histone H2A or a variant phosphorylated close to its *C*-terminus, and the altered chromatin structure associated with γH2A-foci is thought to facilitate repair (Glover and Horn, 2012). Surprisingly, a break immediately adjacent to telomeric repeats fails to trigger the G_2_/M checkpoint and often led to BES deletion. Similarly deprotected telomeres have also recently been shown to fail to contribute to the G_2_/M checkpoint in mammalian cells (Cesare *et al*., 2013) and this may reflect the presence of a distinct chromatin structure at telomeres. Taken together, these results indicate that the DSB response and frequency and mechanism of antigenic variation are highly dependent upon the position of a break within the BES (Glover *et al*., 2013).

Fundamental to HR is Rad51, a homologous-strand exchange enzyme. *T. brucei* RAD51 (McCulloch and Barry, 1999) and a RAD51 paralogue, RAD51-3 (Proudfoot and McCulloch, 2005; Dobson *et al*., 2011), are important for HR, the DSB response and *VSG* switching. Knockout of the *T. brucei* orthologue of BRCA2, required to load RAD51 onto single-stranded DNA during repair, also reduces the *VSG* switching frequency to a level similar to that seen in RAD51 and RAD51-3 mutants (Hartley and McCulloch, 2008). In contrast, *VSG* gene conversion frequency increases in TOPO3α topoisomerase (Kim and Cross, 2010) and RMI1 mutants (Kim and Cross, 2011). These results suggest that a conserved TOPO3α-RMI1 complex, required to control mitotic crossover, promotes *VSG* switching via 70 bp repeat-initiated recombination, while reducing recombination elsewhere along the BES (Kim and Cross, 2010; 2011[Bibr b47],[Bibr b48]). Compared with BRCA2 in other eukaryotes, *T. brucei* BRCA2 has undergone a striking expansion in the number of BRC repeats, the number of which correlates with the efficiency of HR and RAD51 loading (Hartley and McCulloch, 2008); this may facilitate antigenic variation and long-term immune evasion (Trenaman *et al*., 2013).

Although RAD51-dependent mechanisms of *VSG* switching predominate, RAD51-independent mechanisms also operate (McCulloch and Barry, 1999). In *T. brucei*, microhomology-mediated end-joining (MMEJ) is the predominant RAD51-independent pathway, which uses 5–25 bp of imperfectly matched sequence to repair DSBs (Conway *et al*., 2002b; Glover *et al*., 2011). In other systems, non-homologous end-joining is the favoured Rad51-independent form of repair; however, *T. brucei* lacks key components of this pathway. Given the important role of tracts of imperfect 70 bp repeats in *VSG* recombination reactions (Fig. 1B), MMEJ could make an important contribution to *VSG* switching (Glover *et al*., 2011).

## DNA replication and the heritability of *VSG* expression

A number of studies are providing insights into the machinery, co-ordination and regulation of *T. brucei* nuclear DNA replication and suggest a link with antigenic variation. The first link between the DNA replication machinery and *VSG* transcriptional control was based on studies of *T. brucei* ORC1/CDC6 (Godoy *et al*., 2009), a factor related to the Orc1 subunit of the conserved eukaryotic Origin Recognition Complex (ORC) and to Cdc6, which mediates ORC interaction with the replicative minichromosome maintenance (MCM) helicase. Knockdown of ORC1/CDC6 derepresses metacyclic *VSGs* in insect-stage cells (Tiengwe *et al*., 2012) and BESs in bloodstream-form cells and, to a lesser extent, BESs in insect-stage cells (Benmerzouga *et al*., 2013). Transient ORC1/CDC6 knockdown also increases BES switch frequency by approximately threefold (Benmerzouga *et al*., 2013). A genetic screen has revealed some loss of silencing at BESs in insect-stage cells and loss of silencing at BESs and *procyclin* loci in bloodstream-form cells following knockdown of MCM-binding protein (Kim *et al*., 2013). This factor appears to constitute a variant of the replicative MCM helicase, though a direct role in *T. brucei* replication has yet to be demonstrated. These findings implicate DNA replication in *VSG* transcriptional control, but the basis of this, and in particular whether there is a common mechanistic action of ORC1/CDC6 and MCM-binding protein, remains unclear. Association of ORC1/CDC6 with telomeres (Benmerzouga *et al*., 2013) may explain the observed effects, since Orc1 plays a role in gene silencing in yeast, *P. falciparum* and other organisms, both at telomeres and elsewhere (Sasaki and Gilbert, 2007). However, *T. brucei* ORC1/CDC6 is remarkably small relative to Orc1 orthologues and lacks the *N*-terminal bromo-adjacent homology domain involved in binding HP1, which acts in heterochromatin-mediated silencing in other organisms (Flueck *et al*., 2009; Perez-Toledo *et al*., 2009).

Two less direct explanations for the roles of ORC1/CDC6 and perhaps MCM-binding protein in *VSG* expression are also possible. Loss of ORC1/CDC6 is likely to reduce the number of replication origins and hence replication efficiency, which in turn is likely signalled by the cell-cycle checkpoint machinery; in other eukaryotes ORC mutations trigger a Rad9-dependent checkpoint, arresting cells in S-phase (Ide *et al*., 2007). One genomic feature that Rad9 detects is methylation on H3K79, a modification catalysed by Dot1 (Nguyen and Zhang, 2011). In *T. brucei*, the equivalent histone residue, H3K76, is di-or tri-methylated by two enzymes, DOT1A and DOT1B, respectively (Janzen *et al*., 2006b). Although only DOT1A is linked to replication (Gassen *et al*., 2012), the alterations in *VSG* transcriptional control and *VSG* switching dynamics present in *dot1b* mutants, may be due to a link between replication and checkpoint signalling (Stockdale *et al*., 2008). Alternatively, the observed effects of the knockdowns on *VSG* expression and switching may relate to chromosome dynamics after replication. ORC in other eukaryotes is implicated in sister chromatid cohesion; pre-replication complexes can direct loading of cohesin, and ORC provides a cohesin-independent route for sister chromatid association in budding yeast (Diaz-Martinez *et al*., 2008). Thus, the effects of *T. brucei* cohesin knockdown, which also causes elevated BES switching (Landeira *et al*., 2009), may have a basis in interactions between sister chromatid cohesion and replication.

Other studies also suggest that DNA replication acts in antigenic variation. The DNA DSBs found within the *VSG* BESs (Boothroyd *et al*., 2009; Glover *et al*., 2013) may form following replication stalling and replication-fork collapse. In addition, inheritance of the active and silent *VSGs* in their previously transcribed or silent states clearly depends upon the replication process. However, mechanistic data are currently lacking here, and we do not yet know the timing, rate or direction of replication through BESs or other *VSG* loci.

## Conclusions

The available evidence indicates that *VSG* allelic exclusion and recombination, both essential aspects of antigenic variation in *T. brucei*, are critically dependent upon the telomeric environment. Emerging evidence also reinforces the importance of distinct chromatin territories within the nuclear space, although cause or consequence is less certain here. The sub-telomeric context likely provides an environment that experiences more frequent breaks, which allowed *T. brucei* to effectively co-opt and potentially modify a natural response to DNA breaks to achieve efficient antigenic variation. These typically heterochromatic loci also facilitated the massive expansion of the *VSG* gene family without multi-gene expression. Modifications have been achieved through exploiting minichromosomes to increase the maximum telomeric *VSG*-count by approximately 10-fold, by expansion of recombinogenic 70 bp repeats flanking *VSG* genes and, potentially, through BRC-repeat expansions within BRCA2. Clusters of large numbers of silent telomeric *VSGs* likely now facilitate the homology search and improve access to templates for repair. *T. brucei* has also co-opted the Pol-I machinery for *VSG* expression, leading to the formation of a novel extranucleolar, telomere-associated Pol-I compartment.

There are a complex variety of chromatin states that could impact transcription, recombination and replication at *VSG* loci, and it will be important to determine the *cis*-regulatory sequences, the *trans*-acting factors and how they interact to drive *VSG* exclusion and switching. An approach focussed on *T. brucei* homologues of DNA repair, transcription regulatory and chromatin-associated factors identified in other systems has been fruitful. However, an important goal for the future is to seek factors that play more direct and specific roles in *VSG* expression control, some of which may represent exploitable drug-targets. Such factors should further illuminate the mechanisms underlying monotelomeric *VSG* expression and recombination, the processes that make *T. brucei* such a persistent parasite.
